# Lévy random walks on multiplex networks

**DOI:** 10.1038/srep37641

**Published:** 2016-11-28

**Authors:** Quantong Guo, Emanuele Cozzo, Zhiming Zheng, Yamir Moreno

**Affiliations:** 1 School of Mathematics and Systems Science, Beihang University, Beijing 100191, China; 2 Key Laboratory of Mathematics Informatics Behavioral Semantics(LMIB), Ministry of Education, China; 3 Institute for Biocomputation and Physics of Complex Systems (BIFI), University of Zaragoza, Zaragoza 50018, Spain; 4 School of Mathematical Sciences, Peking University, Beijing 100191, China; 5 Department of Theoretical Physics, University of Zaragoza, Zaragoza 50009, Spain; 6 Complex Networks and Systems Lagrange Lab, Institute for Scientific Interchange, Turin, Italy

## Abstract

Random walks constitute a fundamental mechanism for many dynamics taking place on complex networks. Besides, as a more realistic description of our society, multiplex networks have been receiving a growing interest, as well as the dynamical processes that occur on top of them. Here, inspired by one specific model of random walks that seems to be ubiquitous across many scientific fields, the Lévy flight, we study a new navigation strategy on top of multiplex networks. Capitalizing on spectral graph and stochastic matrix theories, we derive analytical expressions for the mean first passage time and the average time to reach a node on these networks. Moreover, we also explore the efficiency of Lévy random walks, which we found to be very different as compared to the single layer scenario, accounting for the structure and dynamics inherent to the multiplex network. Finally, by comparing with some other important random walk processes defined on multiplex networks, we find that in some region of the parameters, a Lévy random walk is the most efficient strategy. Our results give us a deeper understanding of Lévy random walks and show the importance of considering the topological structure of multiplex networks when trying to find efficient navigation strategies.

The study of networks has experienced a burst of activity in the last two decades^1^,^2^,^3^,^4^. Many diverse dynamical processes have been explored on top of networks, including diffusion processes^5^,^6^,^7^, synchronization^8^,^9^, percolation^10^,^11^, to cite just a few^12^. Among these dynamical processes, owning to their wide applications in many scientific fields, including financial time series analysis^13^, social sciences^14^, genetics^15^ among others, random walks have been attracting more and more attention^16^,^17^,^18^,^19^,^20^,^21^. Random walks can be used to study transport and to develop different sorts of searching algorithms on networks, with the aim of finding optimal navigation strategies^22^,^23^,^24^,^25^. A diversity of random walk processes can be defined and studied, however, most of them rely on the classical random walk process whose dynamics occurs according to the topology of the network^21^. In the later scenario, the random walker can only hop to one of the nearest neighbours of the node where it is at any given time, with some -generally the same- probability. Another common random walk process, named Lévy flight, represents the best strategy for randomly searching a target in an unknown environment. This latter kind of random walk dynamics has been widely observed in many animal species^26^,^27^. In its simplest schematization, this stochastic process could drive a walker over very long distances in a single step event that is called ‘flight’^28^. The length of the jump, *l*, obeys a power-law probability distribution in the form of *P*(*l*) ~ *l*^−*α *^26, which makes it possible for the random walker to hop from one node to any other node.

On the other hand, multiplex networks^29^,^30^,^31^,^32^, i.e., networks composed by many different layers of interactions, are gaining much attention recently. The social and technological revolution brought by the Internet and mobile connections, chats, on-line social networks, and a plethora of other human-to-human machine mediated channels of communications have revealed the need to consider that networks might be made up by many different layers of interactions. The same occurs in other fields, like in contemporary biology, where the needs to integrate multiple sets of omic data naturally leads to a multiplex network as a schematization of the system under study. Also in the traditional field of transportation networks, the concept of multiplex networks has a natural translation in different modes of transportations connecting the same physical location in a city, a country, or on the globe. Finally, in the area of engineering and critical infrastructures, it applies to the interdependence of different lifelines. Furthermore, research shows that the topological and dynamical properties of a multiplex network are in general different as compared to those of a single layer network^33^,^34^,^35^,^36^,^37^, as well as the dynamical processes on it^38^,^39^,^40^. For example, it has been shown that a diffusion process can have an enhanced-diffusive behaviour^7^ on a multiplex network, which means that the time scale associated to it is shorter than that occurring on a single layer network.

All the already existing studies of random walks on multiplex networks adopt a nearest-neighbour navigation strategy^41^,^42^. The aim of this paper is to generalize Lévy flights random walks to multiplex networks, which means that a random walker has a certain probability to move to any other node without the need of a direct connection as far as the network is concerned. At each step, the random walker has three options: the first one is to stay at the same node, the second one is to jump to other nodes on the same layer and the last one is to switch to one of its counterparts on other layers, as illustrated in Fig. 1. According to the definition of the dynamics, we obtain the expression for the stationary distribution and the random walk centrality^21^. Besides, with the help of stochastic matrix theory^17^,^43^, we derive the exact expression for the mean first passage time (MFPT). The MFPT is used to describe the expected time needed for a random walker starting from a source point to reach a given target point^44^. Finally, we also compare the results for Lévy flights with other random walks dynamics obtained also on multiplex networks^41^, finding that, under certain conditions, the Lévy random walk is the most efficient from a global viewpoint.

## Results

In this work we consider undirected connected node-aligned multiplex networks^30^. A node-aligned multiplex network is made up of *L* layers with N nodes *i* = {1, 2, …, *N*} on each layer. An adjacency matrix 
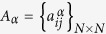
, with *α* = {1, 2, …, *L*}, is associated to each layer *α*. Besides, a coupling matrix 
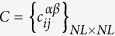
 describes the coupling between nodes in different layers; since each node is coupled only with its counterparts in different layers, then, only the elements of the type 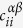 are different from zero.

The whole multiplex network can be described by the supra-adjacency matrix 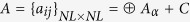
30. Additionally, we consider another set of matrices associated with the multiplex network, that is, we consider a distance matrix *D*_*α*_ = {*d*^*α*^}_*N*×*N*_ associated to each layer *α*, where the element 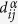 encodes the length (number of steps) of the shortest path connecting node *i* to node *j* in layer *α*^26^. We indicate the probability to find a random walker in node *j* of layer *β* at time *t* starting from node *i* of layer *α* at *t* = 0 by 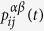. The discrete-time master equation is given:


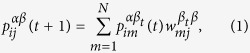


where 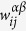 is the transition probability of moving from node i of layer *α* to node j of layer *β*.

To account for the inter-layer connections, we introduce 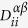 to quantify the “cost” to switch from layer *α* to layer *β* at node i, while 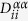 quantifies the “cost” of staying in the same node and in the same layer.

We can now define the transition probabilities 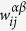 to be


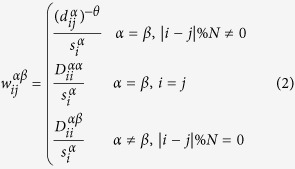


where 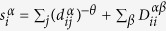 is the strength of node *i* with respect to its connections in the multiplex network, which takes into account the probability of staying at this node and of switching to another layer. As in the case of traditional single layer networks^26^, the transition probabilities 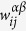 define a dynamical process where a random walker can visit not only the nearest neighbours of a node, but also nodes without direct connections with it on the same layer, while the random walker can switch layer only staying at the same node. Since 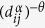 has an exponential decay according to the shortest path between the source node and the target node, the farther they are, the smaller the probability to hop to one from the other. The parameter *θ*, which varies in the range [0, ∞), controls the decay of this probability.

In the following we will derive the mean first passage time (MFPT), that is a characteristic quantity related to a random walk^17^. By iterating Eq. (1), we get an explicit expression for 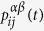:


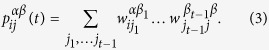


Comparing 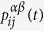 with 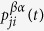 according to the definition in Eq. (2), we get


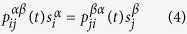


For the stationary solution, which corresponds to the infinite time limit, we can get 
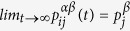
26. Hence, Eq. (4) implies that 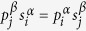 and the probability 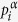 reduces to


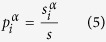


where 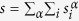 characterises the strength of the whole multiplex network. The expression of the stationary distribution 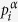 shows that the larger the strength of node *i*, the more often it will be visited, which is valid for any undirected network^45^.

The average of the MFPT over the stationary distribution is (see Methods for details of the derivation)


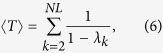


whew *λ*_*k*_ are the eigenvalues of the matrix 
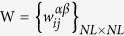
, with 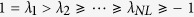. Besides, the random walk centrality of node *i*, as introduced in ref. 21, is 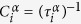, where 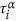 is defined as 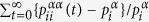. 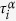 is given by (see Methods)


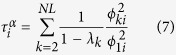


Hence, we have derived the exact expression of the transition probability 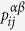 and the MFPT 〈*T*〉. In addition, in order to analyse the navigation of Lévy walks, we average all the 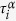
’s over the whole network, which means 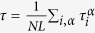. Note that with respect to the local index *τ*_*i*_, which represents the average time needed to reach node *i* from a randomly chosen node, *τ* gives the average number of steps needed to reach any node independently of the initial condition^26^.

**Figure 2:**
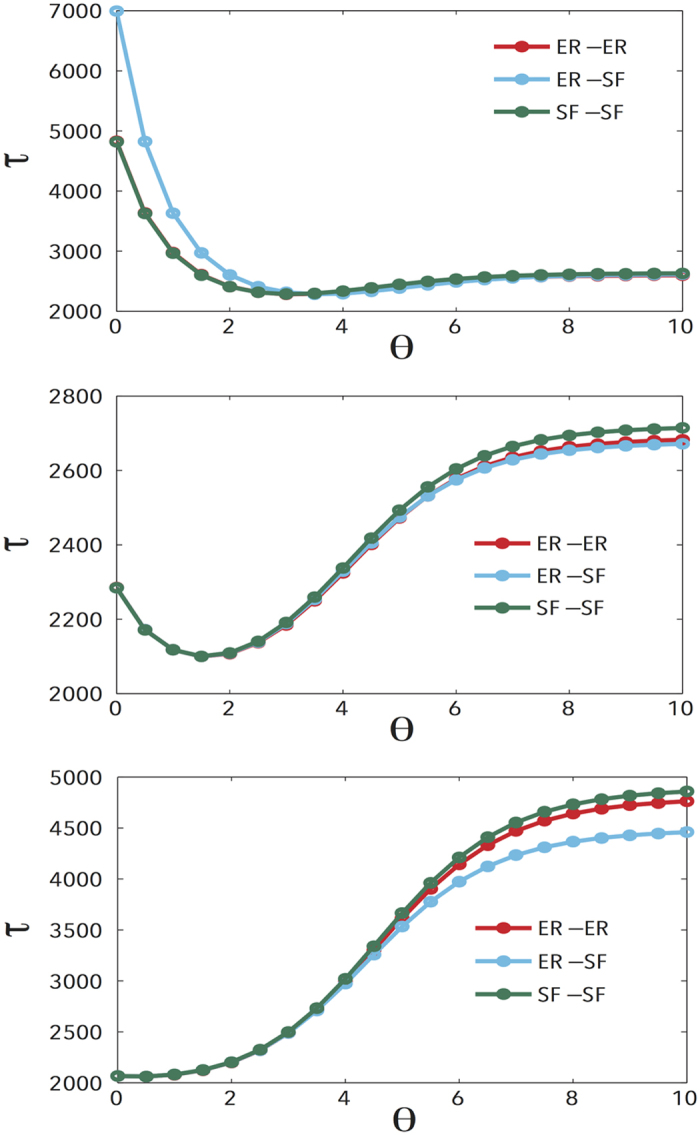
The quantity *τ* vs the Lévy flight index *θ* for different two-layer multiplex networks. Each layer is an ER network or a SF network with 1000 nodes as indicated in the legends. The values of the coupling strength *D*_*X*_ between the two layers are: (**a**) *D*_*X*_ = 0.1, (**b**) *D*_*X*_ = 1, (**c**) *D*_*X*_ = 10.

Next, we proceed to characterise Lévy random walks on multiplex networks drawing on the exact analytic results given above. For the sake of simplicity, following ref. 41, we assign the same value *D*_*X*_ to all the 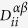, i.e., switching layers has the same cost at any node. In Fig. 2 we show *τ* vs *θ* for different topologies and different values of the cost *D*_*X*_. It is worth noting that, while for large values of the parameter *D*_*X*_ the behaviour of the time *τ* when varying *θ* is qualitatively similar to the classical case of single layer networks, for small and intermediate values of *D*_*X*_ it deviates significantly from the classical case. In particular, the relationship between *τ* and *θ* appears to be of three different kinds depending on *D*_*X*_: when *D*_*X*_ is sufficiently small (*D*_*X*_ = 0.1 in panel (a)) *τ* decreases quickly for small value of *θ*, while it remains more or less constant for large values of *θ*, when *D*_*X*_ is sufficiently large (*D*_*X*_ = 10 in panel (c)) *τ* increases monotonically with *θ*, as in classical single layer networks, with the speed of the increase being much smaller when *θ* is small. Furthermore, for intermediate values of *D*_*X*_ (*D*_*X*_ = 1 in panel (b)) *τ* shows a clear minimum for a given value of *θ*. This phenomenology, that is, the fact that the efficiency depends on the coupling, constitutes the central finding of our study.

In single layer networks, setting *θ* = 0 is always the best strategy to navigate a network, as the global time *τ* is minimum. However, in multiplex networks the value of *θ* -it’s optimal- that minimizes *τ* depends on the value of the coupling *D*_*X*_. Interestingly enough, for low values of *D*_*X*_, the limiting case *θ* → ∞, which corresponds to the normal random walks on networks, can be more efficient than *θ* = 0. Other scenarios worth inspecting are given by the topologies of the networks that made up each layer of the multiplex. In particular, a multiplex network can be made up of different combinations of homogeneous (Erdos-Renyi (ER)) and heterogeneous (Scale-Free (SF)) networks. We have also explored these scenarios numerically for different regimes of the coupling parameter. For *D*_*X*_ ≪= 1, different structures lead to different relationships between *τ* and *θ*. When *θ* is small, a SF-SF multiplex network has a much smaller *τ* than an ER-ER or an ER-SF multiplex network; however, if *D*_*X*_ is bigger than 1, the difference is evident only when *θ* ≫ 0. Altogether, the previous results show that whether the optimal value of the Lévy walk index *θ* is constant across different multiplex topologies depends on the value of the coupling strength *D*_*X*_.

In our model *τ* attains its minimum at a non-zero value of *θ*, as shown in Fig. 2, while in the case of Lévy random walks in single layer networks *τ* always gets its minimum at *θ* = 0, i.e., *τ* is a monotone increasing function of *θ*^26^. This indicates, as can be seen in Eq. (2), that there exists competition and balance between two different dynamics: the random walk inside a given layer and the random switching between layers. Note that *τ* gives the average number of steps needed to reach any node independently of the initial condition. Hence, considering a multiplex network as a whole, it’s crucial for the random walker to efficiently jump between different layers to efficiently cover all the nodes. Taking the definition in Eq. (2) into consideration, we classify three situations according to the values of *D*_*X*_:

- *D*_*X*_ ≪ 1. In this case, when *θ* is too small, it is difficult for the random walker to switch to other layers, then, compared with a single layer setting, the value of *τ* becomes larger at small values of *θ*. Especially if *θ* = 0, 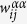 stays the same while 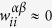, that is the reason why at this point *τ* attains a much bigger value.
- *D*_*X*_ = 1. In this case, when *θ* is too large, which almost corresponds to normal random walks on networks, although the switching between layers becomes easier, without the ‘small world’ effects of Lévy random walks^26^, the process is much more inefficient. However, if *θ* = 0, since *D*_*X*_ = 1 and 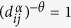, the counterpart of a node on another layer is just like one of its neighbors in the current layer. The fact that *τ* is still not at its minimum implies that the transition probability between layers should be larger compared with that on the same layer. This addresses the importance of the counterpart of a node, because it is the only way for random walkers to visit another layer.
- *D*_*X*_ ≫ 1. Just as described in the case *D*_*X*_ = 1, the counterpart should be treated not only as a normal neighbor to make *τ* to be the minimum. In this case, *D*_*X*_ ≫ 1 indicates that the transition probability between a node and its counterpart is the largest one since on the same layer 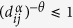. Hence, *τ* has its minimum around *θ* = 0.

From the discussion above, the fact that *τ* has its minimum at a non-zero value of *θ* is thus a result of the competition and balance of the two different underlying dynamics. Among these dynamics, whether the transition probability between layers is the larger one compared with that between nodes on the same layer is the important characteristic. These results also further explain the effects of the topology of multiplex networks on random walk processes.

**Figure 3:**
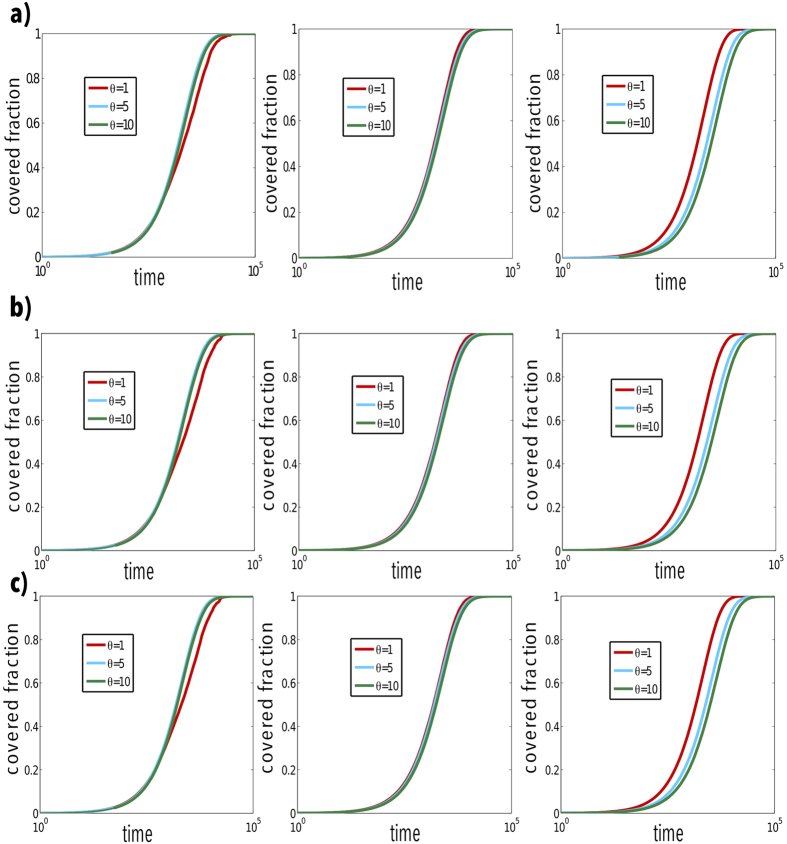
Number of visited nodes versus time for Lévy random walks on multiplex networks. The structures of the multiplex networks considered are: ER-ER (top panels a), ER-SF (middle panels b) and SF-SF (bottom panels c). In each configuration, the synthetic networks of each layer are composed of 10^3^ nodes. From left to right, the values of *D*_*X*_ are 0.1, 1, 10. The Lévy index *θ* used are indicated in the legend.

Next, in order to provide more numerical evidences of what we have found analytically, we present the results obtained when the fraction of covered nodes is taken into consideration. Figure 3 shows this magnitude as a function of time for different multiplex networks. In the first case (panel a), the network is made up of two ER networks with the same structure. For a small value of *D*_*X*_ (upmost left figure), it can be seen that the bigger the value of *θ* is, the higher the efficiency of the Lévy random walk is. This also confirms the results in Fig. 2, since a larger value of *θ* leads to a smaller value of *τ*. With respect to other values of *D*_*X*_, such as *D*_*X*_ = 1 (middle figures in all panels), *D*_*X*_ = 10 (upmost right figures in all panels), the results for *τ* in Fig. 2 are also confirmed. In the case of other kinds of arrangements for the networks at each layer, we obtain the same results, as can be seen from panels b and c in Fig. 3. Furthermore, the comparison of the results obtained for different combinations shows that the topologies of the networks in each layer do not play a significant role.

**Figure 4:**
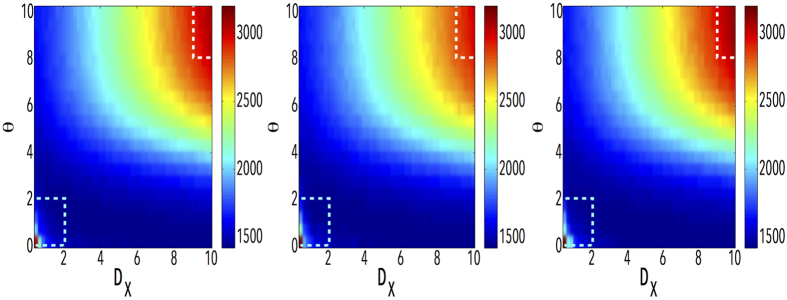
The effects of inter-layer weight *D*_*X*_ and Lévy index *θ* on the efficiency of Lévy random walks. The color-coded map describes the time needed to cover 50% of all the nodes (10^3^) in each layer. From left to right, the structure of network in each layer is ER-ER, ER-SF and SF-SF, respectively. The rectangles highlights the areas that show the largest differences due to the multiplex structure of the system.

**Figure 5:**
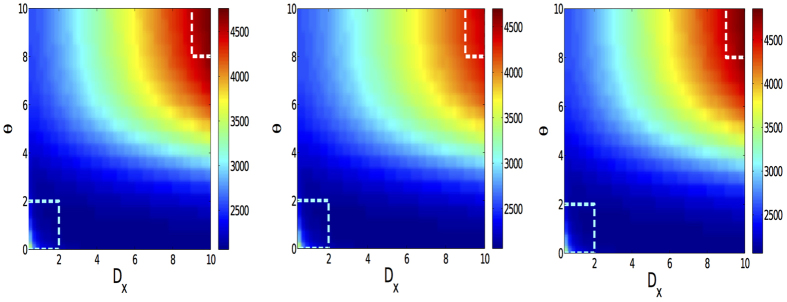
The behavior of *τ* as a function of *θ* and *D*_*X*_. From left to right, the structure of network in each layer is ER-ER, ER-SF and SF-SF, respectively. The rectangles highlight the areas that show the largest differences due to the multiplex structure of the system.

The previous results indicate that the coupling strength between layers is a crucial factor determining the structure and the dynamical behavior of the system^46^. In addition, as described above, being used to characterize the cost for a walker to switch between layers, the value of *D*_*X*_ also has distinct effects on Lévy random walks on multiplex networks. In order to further explore the details of these effects, as a function of *θ*, we show in Fig. 4 the time needed to cover the 50% of all nodes as a function of both *D*_*X*_ and *θ*. As shown in the figure, an interesting phenomenology appears. Firstly, the highest values of the time needed to cover half of the network locate at the up-right and down-left corners, where the values of *D*_*X*_ and *θ* are the biggest and the smallest. Moreover, in a significant range of values of *θ*, increasing *D*_*X*_ does not change greatly the time needed to cover 50% of the nodes. However, if *θ* is large enough, the increase of *D*_*X*_ have a larger impact. Note that these results also confirm the analytical findings about *τ*, as can be seen in Fig. 2. Besides, we have also explored the dependence of *τ* on *D*_*X*_ and *θ* in Fig. 5. Just like the fraction of visited nodes, the highest values of *τ* locate at the up-right and down-left corners.

Another result worth highlighting that connects our results for *τ* with the structural properties of the multiplex involves the second largest eigenvalue *λ*_2_. As shown for the smallest eigenvalue of the supra-Laplacian^46^, there is a transition point that separates two different regimes in interdependent networks: in one regime, all the layers are structurally decoupled and in the other regime, the system behaves as a single layer. The same result holds for the second smallest eigenvalue of the generalized supra-Laplacian of a multiplex network when increasing the coupling strength *D*_*X*_. Specifically, the generalized supra-Laplacian is


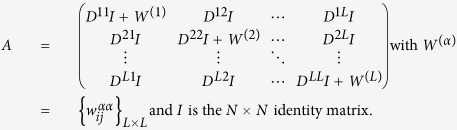


**Figure 6:**
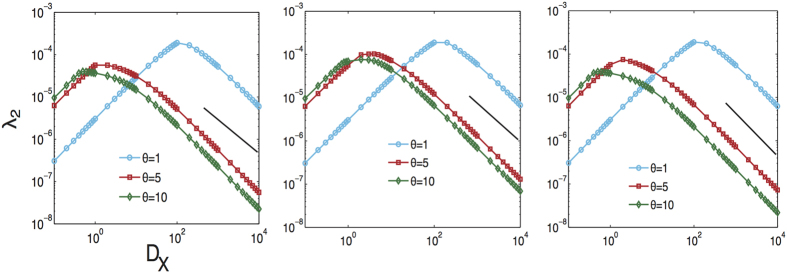
The second smallest eigenvalue of the generalized supra-laplacian matrix as a function of the inter-layer weight *D*_*X*_ for three different multiplex topologies, which from left to right are ER-ER, ER-SF, SF-SF, respectively. Each panel describes Lévy random walk with different Lévy index *θ* (1, 5, 10). The solid line corresponds to 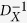.

Figure 6 shows the dependency of *λ*_2_ with *D*_*X*_ for different values of the Lévy flight parameter Θ. Also in this case (see ref. 41), 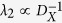, regardless of the network structure as showed it can be seen in the different panels of Fig. 6. Finally, for the sake of comparison with results obtained for other random walk dynamics, we compare their efficiency^41^ with that of the Lévy flight. In the first case (RWC), the random walker in node *i* can move to any one of its neighbors *j* on the same layer with the transition probability 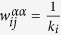, where *k*_*i*_ is the degree of node *i*. Secondly, we also consider the case (RWD) in which the random walker is allowed to jump to any other node with probability 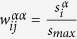, where 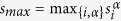. Lastly, a third scenario (RWP) considers that it is possible for a random walker to switch layers and jump to another neighborhood at the same step.

**Figure 7:**
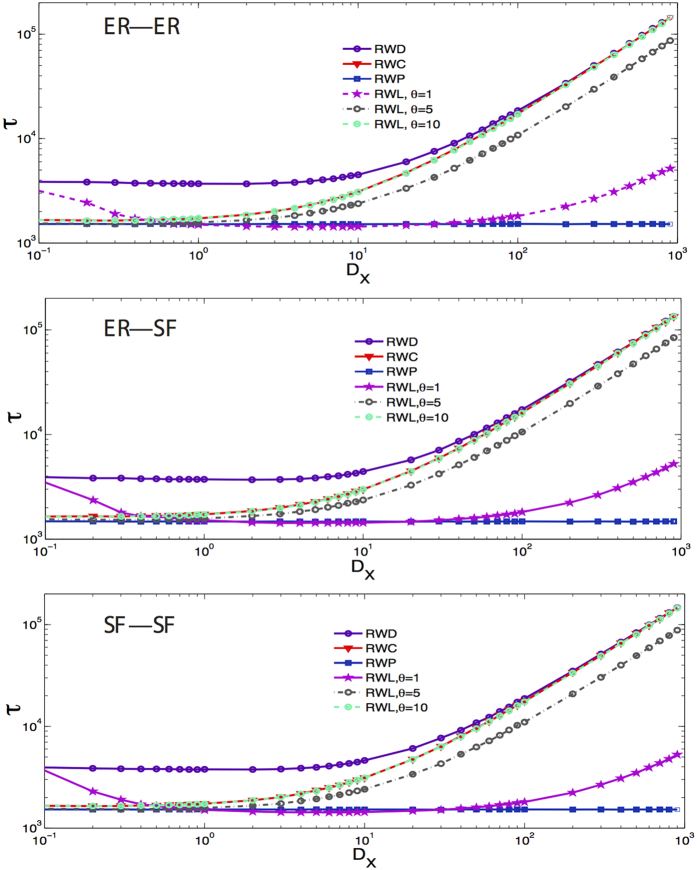
Time needed to cover 50% of all nodes on three types of multiplex networks, as a function of the inter-layer weight *D*_*X*_. We compare the results obtained for the Lévy random walk (RWL) studied here with three other scenarios for the walks, as discussed in the text. The values of *θ* considered are 1, 5, 10, respectively. Each layer has 10^3^ nodes and all the simulations were averaged over 100 realizations.

In Fig. 7, we show the time *τ* needed to cover 50% of all the nodes as a function of the value of *D*_*X*_ with respect to different topological structures. For the Lévy random walk, we study three different cases where the index *θ* equals 1, 5, 10, respectively. Comparing the Lévy case with the three others mentioned above, one can get further insights on the different strategies for navigation, that is to say, there is no strategy that is always the most efficient for any network and an arbitrary coupling strength. For instance, taking the Lévy random walk as an example: when *θ* is small (*θ* = 1), the time *τ* appears to be the smallest in the range 1 < *D*_*X*_ < 10, but as *θ* if further increased, the time needed to cover the 50% of the nodes of the network is almost the same as compared to that needed for a classical random walk, which in its turn is not the most efficient. This is easy to understand because in a Lévy random walk, when *θ* → ∞, the transition probability 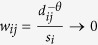 if the shortest path *d*_*ij*_ is larger than 1. Therefore, the fist of the cases to which we compare -i.e., the classical random walk- is a special case of a Lévy walk when *θ* → ∞^26^.

Furthermore, in order to explore the behavior of *τ* as a function of *D*_*X*_ in the studied Lévy walk model and other random walk models, we summarize the transition rules for these different kinds of random walk processes in a multiplex network in Table 1. Note that in these expressions, 
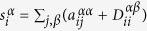
 and 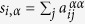, 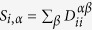, 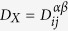
41, where for RWC, RWD and RWP models, if there is a link between node *i* and *j* of layer *α*, 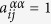 else 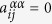. Then, for the RWP model, since all the values of 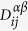 are the same, it is known that *τ* is the same for any value of *D*_*X*_. Besides, for the RWD model, if *D*_*X*_ is large enough, the main transition activity of random walkers is to jump among different counterparts of the same node instead of exploring their neighbors on the same layer, which leads to larger values of *τ*. For the Lévy walk model (RWL), since it has another parameter *θ*, we should consider different values of *θ* for this model. When *θ* is small, just as discussed in Fig. 2, small *D*_*X*_ leads to large *τ* and much larger *D*_*X*_ also leads to the increase of *τ*. While if *θ* is large, only if *D*_*X*_ is also large *τ* can be large since it takes too many steps for the random walkers to switch among the counterparts of a node. In the last scenario, for the RWC model, due to the fact that it is a special case of a Lévy walk when *θ* → ∞, the behavior of *τ* as a function of *D*_*X*_ is the same of that of the RWL model with large enough *θ*.

## Discussion

In summary, we have studied Lévy random walks on multiplex networks. With the help of stochastic matrix theory, we have calculated analytically the expression of the stationary distribution and MFPT from any node to any other node. Besides, we have also obtained an exact expression of the average time *τ* needed to reach a node regardless of the source node. This dynamics on multiplex networks shows a strong dependence on the inter-layer weight *D*_*X*_ and the Lévy index *θ*. Our main result is that when *D*_*X*_ is small enough, contrary to the case of a Lévy random walk on single layer networks, the bigger the index *θ* is, the more efficient the Lévy random walk is. In order words, in that region of parameter values, although it is not very likely for any given walker to jump directly to other nodes far away, the total average time *τ* needed to visit any node independently of the initial condition is smaller. Interestingly, if the value of *θ* is not too large, for instance for *θ* < 4, *D*_*X*_ does not have a significative impact on *τ*. The present results add to previous works that explored other kinds of random walk processes on multiplex networks, and allow to have a more complete picture that highlights the importance of considering the interconnected nature of many systems if we aim at finding the best navigation strategies and develop searching and navigability algorithms for such interdependent networked systems.

## Methods

In the following, by using the formalism of generating functions^47^, we will get the analytical result for MFPT. The first passage probability 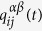 from node i of layer *α* to node j of layer *β* after *t* steps satisfies the relation


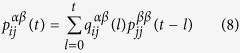


Let 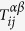 denote the MFPT from node i of layer *α* to node j of layer *β*, then


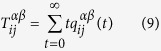


here, as proposed in ref. 17, we introduce the following generating functions:


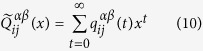



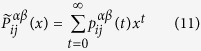


where |*x*| < 1, inserting Eq. 8 into 11, we get


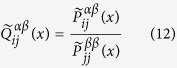


Since 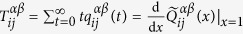, the problem of solving for the MFPT is reduced to calculate the derivative of 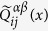 and evaluate it at *x* = 1.

We will address this point making use of the stochastic matrix theory^43^. For the sake of simplicity, we use the matrix 
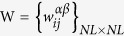
 and the matrix 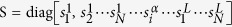 to describe the transition probabilities and node strengths, respectively. It is clear that the matrix *W* is a stochastic matrix, since for any node *i* its elements satisfy that 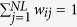. *W* is an antisymmetric matrix. Because of that, we introduce the matrix


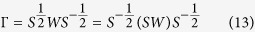


which is symmetric and similar to W. Thus, they have the same eigenvalues. Since Γ can be diagonalized and the eigenvalues are all real, we define 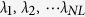 as its eigenvalues. These eigenvalues satisfy that 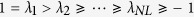. Let 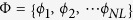 denote the corresponding normalized, real-valued, and mutually orthogonal eigenvectors. As a result, the matrix Γ can be written as


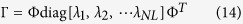


which, together with 13, leads to


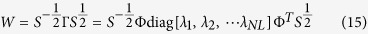


Then considering the master equation Eq. (1), we can get 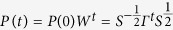, whose element denoted by 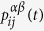 represents the transition probability from node i of layer *α* to node j of layer *β* in t steps. Note that the elements of the matrix *P*(0) fulfill the following relations


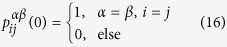


Then, inserting Eq. 15 into the expression of *P*(*t*), one has


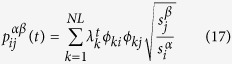


where 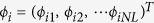 and they satisfy 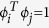 if *i* = *j*, else *ϕ*_*i*_*ϕ*_*j*_ = 0, which means


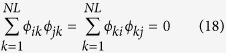


We have now an expression for 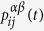, plugging it into Eq. 11, it is easy to obtain


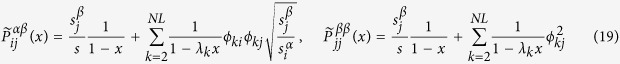


According to the definition given above, the MFPT 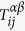 can be calculated by differentiating 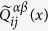



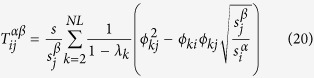


In addition, using Eq. 20, we have


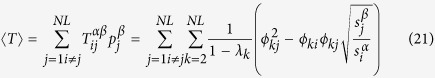


Using Eq. 18 and the relation 
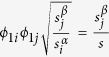
, which means 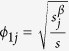, we can get


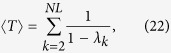


where the time 〈*T*〉 is the average of the MFPT over the stationary distribution, obviously it does not depend on i, and it is known as the Kemeny’s constant^26^. Besides, as introduced in ref. 21, we can calculate the quantity 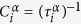, that is the random walk centrality of node *i*, where 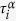 is defined as 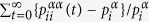. Combining Eq. 17, 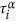 is given by


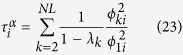


## Additional Information

**How to cite this article**: Guo, Q. *et al*. Lévy random walks on multiplex networks. *Sci. Rep*. **6**, 37641; doi: 10.1038/srep37641 (2016).

**Publisher's note:** Springer Nature remains neutral with regard to jurisdictional claims in published maps and institutional affiliations.

## Figures and Tables

**Figure 1:**
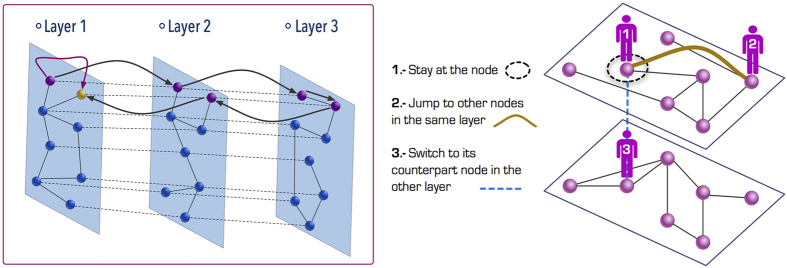
Illustration of the Lévy flight navigation strategy on a multiplex network. In the toy model, we consider a three-layer multiplex network and show two different paths that can lead the walker to the yellow node (one involves a Lévy flight and the other implies that the walker follows the topological path of the graph). The right panel summarizes the different elementary steps that a walker can adopt in our model as indicated.

**Table 1:** Transition probability of four different random walk processes on multiplex networks.

Tr.	RWC	RWD	RWP	RWL
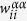	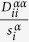	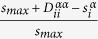	0	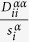
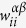	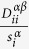	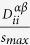	0	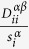
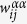	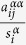	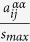	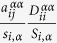	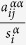
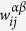	0	0	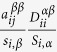	0
